# Effect of manipulation of primary tumour vascularity on metastasis in an adenocarcinoma model

**DOI:** 10.1038/sj.bjc.6600020

**Published:** 2002-01-07

**Authors:** M M Davies, P Mathur, P Carnochan, S Saini, T G Allen-Mersh

**Affiliations:** Division of Surgery, Faculty of Medicine, Imperial College School of Science, Technology and Medicine, Chelsea and Westminster Hospital, 369 Fulham Road, London SW10 9NH, UK; Department of Physics, Institute of Cancer Research, Royal Marsden Hospital, Downs Road, Belmont, Surrey SM2 5PT, UK

**Keywords:** tumour vascularity, metastasis, angiogenesis, basic fibroblast growth factor

## Abstract

One explanation for the clinical association between tumour vascularity and probability of metastasis is that increased primary tumour vascularity enhances haematogenous dissemination by offering greater opportunity for tumour cell invasion into the circulation (intravasation). We devised an experimental tumour metastasis model that allowed manipulation of primary tumour vascularity with differential exposure of the primary and metastatic tumour site to angiogenic agents. We used this model to assess the effects of local and systemic increases in the level of the angiogenic agent basic fibroblast growth factor on metastasis. BDIX rats with implanted hind limb K12/TR adenocarcinoma tumours received either intratumoural or systemic, basic fibroblast growth factor or saline infusion. Both intratumoural and systemic basic fibroblast growth factor infusion resulted in significant increases in tumour vascularity, blood flow and growth, but not lung metastasis, compared with saline-infused controls. Raised basic fibroblast growth factor levels and increase in primary tumour vascularity did not increase metastasis. The clinical association between tumour vascularity and metastasis is most likely to arise from a metastatic tumour genotype that links increased tumour vascularity with greater metastatic potential.

*British Journal of Cancer* (2002) **86**, 123–129. DOI: 10.1038/sj/bjc/6600020 www.bjcancer.com

© 2002 The Cancer Research Campaign

## 

Clinical studies of breast, lung, cervical and colorectal cancer suggest that tumour vascularity is related to risk of metastasis (Matsuyama *et al*, 1998; Nanashima *et al*, 1998; Tjalma *et al*, 1998; Weidner, 1998). One causal explanation is that increased vascularity within the primary tumour may offer greater opportunity for tumour cell invasion into the circulation, resulting in enhanced haematogenous dissemination.

In support of this, a relationship between tumour vascularity and the number of circulating tumour cells (CTC) has been demonstrated in an animal tumour model (Liotta *et al*, 1974). However, more recent clinical studies suggest that CTC's are frequently detected in patients with invasive cancer, regardless of tumour vascularity, stage or clinical outcome (Ennis *et al*, 1997; Wharton *et al*, 1999; Mathur *et al*, 2001). Metastasis involves additional critical steps – including attachment to vascular endothelium, and extravasation (Fidler, 1997) – and the relationship between primary tumour vascularity and metastasis could derive from oncogene mutations that – as in the case of k-ras or p53 – influence vascular (Rak *et al*, 1995; Mehta *et al*, 2001) as well as tumour metastatic (Bartsch *et al*, 1998; Maehara *et al*, 2000) phenotype.

We developed an experimental tumour metastasis model that allowed differential exposure of the primary and metastatic tumour site to the angiogenic agent basic fibroblast growth factor (bFGF), in order to vary primary tumour vascularity without altering tumour genotype. We assessed the effects of variation in tumour vascularity by both local and systemically administered bFGF, on tumour metastasis.

## METHODS

### K12/TR tumour cell line

The K12/TR cell line (courtesy Dr S Watson, Queen's Medical Centre, Nottingham, UK and Dr S Eccles, Institute of Cancer Research, Sutton, UK) is a transplantable hypovascular rat colonic adenocarcinoma that metastasises to the lungs from the subcutaneous implantation site (Dunnington *et al*, 1987).

### *In vitro* K12/TR cell line proliferation in response to bFGF

K12/TR cells suspended in 100 μl DMEM medium were added to 96-well plates, and then incubated overnight at 37°C in 5% CO_2_. The medium was removed and replaced by bFGF diluted in DMEM. Previous *in vitro* studies have demonstrated that a bFGF concentration of 3 ng ml^−1^ induces cell proliferation (Montesano *et al*, 1986). Three bFGF concentrations (3 ng ml^−1^, 30 ng ml^−1^, 150 ng ml^−1^) and a negative (saline) control, were used. The cells were incubated for a further 4 days, and an MTT (3-(4,5-dimethylthiazol)-2,5-diphenyltetrazolium bromide) assay then used to quantify cell number. MTT is a soluble tetrazolium salt that is converted by viable cells to an insoluble formazan precipitate (Mosmann *et al*, 1983) that forms a purple coloured solution when dissolved in an organic solvent. The optical density of the solution is proportional to the number of viable cells (Carmichael *et al*, 1987). 0.1 mg of MTT in 200 ml of PBS was added to each well of the plate which was then incubated at 37°C for 4 h. The medium was then aspirated leaving a formazan crystal residue which was dissolved by adding 200 ml of DMSO to each well and agitating for 10 min. The optical density of each well was read at 570 nm using a spectrophotometer (Titertek Multiscan, Finland).

### *In vivo* K12/TR tumour implantation

The K12/TR cell line was grown and prepared as previously described (Dunnington *et al*, 1987). Male BDIX rats (median weight, 403 g; (range 309–459)) were anaesthetized with a 1–2% mixture of Halothane and oxygen (May & Baker, UK) using a commercial vaporizer (Halovet, IMS, UK). Subcutaneous flank tumours were produced by injecting 0.1 mls phosphate buffered saline containing 10^6^ cells through a cannula placed subcutaneously in the flank of each animal. The animals were then divided into groups for either intratumoural or systemic infusion of either bFGF or saline.

### Osmotic infusion pump insertion and intratumoural bFGF infusion

The flank tumour injection cannula was then connected to an Alzet osmotic mini-pump (Charles River, Maidstone, UK) that was placed in a subcutaneous pocket. The tumour cell inoculum was then continuously infused at a 0.25 μl h^−1^ with either bFGF or saline (control group) for 28 days from the time of cell injection. The bFGF was delivered at a dose rate of 100 ng per 24 h, as this dose has previously been shown to stimulate angiogenesis in a rat subcutaneous sponge model (Hu *et al*, 1994).

### Osmotic infusion pump insertion and systemic bFGF infusion

After flank tumour injection, the internal jugular vein was cannulated and connected to an Alzet osmotic mini-pump (Charles River, Maidstone, UK; 0.25 μl h^−1^). bFGF was delivered at a dose rate of 5 μg per 24 h. This dose has previously been demonstrated to increase liver metastasis vascularity when infused systemically in a rat fibrosarcoma model (Davies *et al*, 2001).

### Flank tumour assessments

Flank tumour blood flow was assessed in non-recovery experiments, after a 4-week tumour growth period (see below).

#### Blood flow

The tissue equilibration method (Tozer and Shaffi, 1993) was used. After anaesthesia for flank tumour excision, 50 μCi of [I-125]-Iodoantipyrine ([I-125]-IAP) in 0.5 ml 0.9% saline was infused over 30 s via a polythene cannula (ID 0.28 mm, Portex, UK) inserted into the internal jugular vein. The infusion was stopped and the animal weighed and killed. The flank tumours were excised, rapidly frozen (<1 min) in a pre-cooled isopentane freezing bath (−70°C), and stored in liquid nitrogen until assessed. Each subcutaneous tumour was bisected and equatorial tissue sections were cut for blood flow (20 μm), vascularity (6 μm) and cell proliferation (6 μm) assessments using a cryostat (Bright Instruments, UK).

Tissue sections for autoradiography were transferred onto glass slides and allowed to dry at room temperature. They were then placed on β-max (Kodak, UK) autoradiography film with calibration standards and then exposed for 3 weeks. Autoradiographic images were digitized for analysis using a PC based image processing system (Microscale TC, Digithirst Ltd, UK), with a pixel resolution of 34 × 38 μm. Line profiles were drawn from the tumour edge to the tumour centre and the transmitted light intensity value of each pixel on the profile was recorded. Each film was calibrated using co-exposed I-125 standards and the light intensity values were converted to (I-125)-IAP concentrations, and then normalized with respect to the tumour edge. Between 6–8 line profiles drawn at intervals around each tumour boundary were averaged per tumour. Tissue concentration of (I-125)-IAP was used as a relative measure of blood flow. We have previously found (unpublished observations) that tissue blood flow is directly proportional to Iodoantipyrine concentration up to flow rates (approximately 0.5 ml min g^−1^) that exceed those reported (Hemingway *et al*, 1991) in the hypovascular tumours used in the present study.

#### Vascularity

Six μm thick cryostat (Bright Instruments, UK) sections were cut and fixed in acetone, and endogenous peroxidase activity was then blocked with 0.1% Hydrogen Peroxide. After washing in TRIS-buffered saline and blocking for non-specific activity with diluted normal rabbit serum (Dako, UK.), sections were incubated with monoclonal Ox-43 primary antibody (MCA 276, Serotec, UK) at a dilution of 1 : 300 for 30 min. Subsequent processing was carried out using the streptavidin-biotin method (Dako) after washing in TBS containing 1 ml 1% 10-ethyl ether. The complex was visualized using diaminobenzydine and counterstained with Mayer's Haemotoxylin. Positive and negative controls were respectively, rat heart muscle, and flank tumour without primary antibody exposure.

After staining, tumour sections were independently assessed for vascularity by two of the authors (MM Davies and P Mathur) at × 400 magnification (Nikon Optiphot, × 10 eye-piece, × 40 objective) without knowledge of infusion group. Vessel volume fraction was assessed by Chalkley's method (Chalkley, 1943) using a 25 dot eyepiece graticule. Vessel length density was assessed by counting all stained features within a 245 μm × 175 μm rectangular field (Aherne and Dunnill, 1982). Forty fields were selected randomly over the tumour section for each measurement. Distribution of vascularity was assessed on line profiles of vessel length density (Underwood, 1970), by counting vessels within adjacent fields while moving from tumour periphery to centre. Between 6–8 such line profiles were obtained and averaged for each tumour section.

#### Proliferation

Proliferation was assessed by quantifying the expression of the 36 kDa nuclear protein Proliferating Cell Nuclear Antigen (PCNA). PCNA is an auxiliary protein for DNA polymerase and it accumulates from stages G1 to M of the cell cycle, its presence within a cell indicates that the cell is proliferating (Hall *et al*, 1990). Detection of the PCNA protein can be achieved by immunohistochemical staining of tumour sections with an anti-PCNA monoclonal antibody (PC10, Dako, UK).

Contiguous 6 μm flank tumour sections were fixed in 4% formalin for 2 min, then ethanol for a further 10 min and blocked for endogenous peroxide activity with 0.1% Hydrogen Peroxide. After washing in TRIS-buffered saline and blocking for non-specific activity with diluted normal rabbit serum (Dako, UK), the sections were incubated with the monoclonal primary anti-PCNA antibody (PC10, Dako, UK) at a dilution of 1 : 100 for 30 min. The secondary and tertiary layers were applied as per the Dako streptavidin-biotin complex after washing in TBS containing 1 ml 1% 10-ethyl ether. The complex was visualized using diaminobenzydine and counterstained with Mayer's Haemotoxylin. Sections were examined at × 400 magnification using a Nikon (Optiphot) microscope (× 10 eyepiece, × 40 objective) with a 245 times; 175 mm rectangular field placed in the eyepiece. One thousand cells were counted in eight randomly placed fields. All brown staining cells were considered positive. PCNA was quantified by the PCNA index which was defined as the percentage of positive staining cells per 1000 cells counted (Teixeira *et al*, 1994).

#### Growth

Every 2 days after tumours became palpable (approximately 14 days after flank tumour injection), two perpendicular (to a vertical axis through the approximate centre of the tumour) measurements were made of the shortest and longest tumour dimensions. A value for the volume of each tumour was then derived according to the formula: Vol=(π/6)×(a^2^×b) (Euhus *et al*, 1986).

### Lung metastasis assessments

After a 4-week primary tumour growth period and a further 8 weeks following flank tumour excision (see above), animals were killed, weighed and examined for lung metastases. Studies in mice suggest that macroscopic examination of lungs for the presence of metastases correlates closely with results of serial sectioning and microscopic examination (Wexler, 1966). Macroscopic examination of lungs was performed to assess the prevalence of metastasis. The number of lung metastases per animal, and lung weight were also assessed as additional indicators of extent of lung metastasis (Kolber *et al*, 1995).

The lungs of each animal were excised and fixed in methacarn (methanol-inhibisitol-acetic acid in the ratio 6 : 3 : 1) overnight and then examined using a dissecting microscope (Nikon Optiphot, Japan; × 10 eye-piece, × 20 objective) for the presence of metastases and weighed. Brain, intra-abdominal organs, and axillary and inguinal lymph node groups were also macroscopically examined for metastases.

### Statistical methods and experimental guidelines

Differences between bFGF and saline infused groups were assessed by Mann Whitney-*U* test. Repeated measures analysis of variance was used to assess vessel length density and tumour:tumour edge ^125^I (blood flow) ratio differences from tumour edge to centre between bFGF and saline infusion groups. Lung metastasis experiments were designed to provide an 80% power of detecting as significant (*P*<0.05), a 50% increase in lung metastasis with the bFGF-induced increase in flank tumour vascularity. This required a total sample of 28 animals in each metastasis experiment.

All experiments using laboratory animals were carried out according to UKCCCR guidelines and were approved under the terms of the UK Home Office Animals (Scientific Procedures) Act 1986. All laboratory animal procedures had also been reviewed by the Royal Marsden Hospital Ethics Committee

## RESULTS

### *In vitro* K12/TR cell line proliferation in response to bFGF

**Figure 1:**
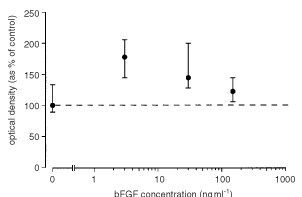
There was a significant increase in proliferation rate of K12/TR cells exposed to bFGF at a concentration of 3 ng ml^−1^ (M.W.U. *P*=0.003) and 30 ng ml^−1^ (M.W.U. *P*=0.01) compared with the negative control.

There was a significant increase in the proliferation rate of K12/TR cells exposed to bFGF at concentrations of 3 ng ml^−1^ (median 200%, iqr 150–250%, *P*=0.003) and 30 ng ml^−1^ (median 133%, iqr 83–167%, *P*=0.01) compared with control (median 100%, iqr 67–183%). Proliferation of cells exposed to bFGF at a concentration of 150 ng ml^−1^ (median 113%, iqr 93–130%) was not significantly different (*P*=0.1) to control (Figure 1

).

### Intratumoural bFGF infusion into K12/TR flank tumours

#### Vascularity, blood flow, proliferation and growth

Ten tumours (five saline and five bFGF) from 10 animals were examined. There was no difference (*P*=0.3) in animal weights in bFGF (371 g (362–421 g)) compared with saline (392 g (362–428 g)) groups.

**Figure 2:**
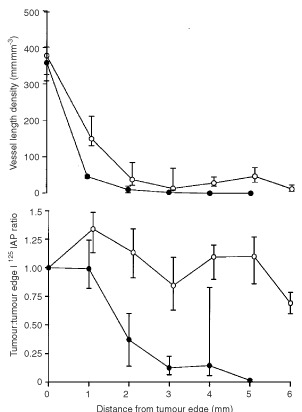
There were significant increases with distance from the tumour edge in vessel length density (repeated measures analysis of variance, *P*=0.0001) (above) and tumour to tumour edge blood flow ratio (*P*=0.0005) (below) in bFGF infused subcutaneous K12/TR tumours compared with controls (•: saline *n*=5; ○: bFGF *n*=5; median and interquartile range).

There was a significant increase (*P*=0.008) in vessel length density in the bFGF (median 65.3 mm mm^−3^, iqr 61.8–77.0 mm mm^−3^) compared with the saline (35.0 mm mm^−3^, 33.8–35.6 mm mm^−3^) groups. Similarly, there was a significant increase (*P*=0.004) in vessel volume fraction in the bFGF (5.9%, 5.8–6.2%) compared with the saline (3.6%, 2.5–4.3%) group. There was also a significant increase (*P*=0.0001) in vessel length density with distance from the tumour edge between bFGF and saline infused tumours (Figure 2

). There was a significant increase in tumour blood flow (*P*=0.0005) between bFGF infused compared with saline-infused tumours (Figure 2).

**Figure 3:**
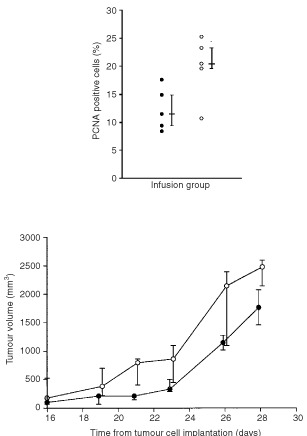
There was a significant increase (M.W.U. *P*=0.03) in the PCNA positive cell index (above) and in the tumour volume (M.W.U. *P*=0.05) (below) of bFGF infused subcutaneous K12/TR tumours compared with controls (•: saline *n*=5; ○: bFGF *n*=5; median and interquartile range).

There was a significant increase (*P*=0.03) in PCNA index in the bFGF (median 20.5%, iqr 19.6–23.3%) compared with the saline (11.5%, 9.4–14.9%) group. This was associated with a significant increase (*P*=0.05) in the volume of bFGF compared with saline infused tumours (Figure 3

).

#### Lung metastasis

**Figure 4:**
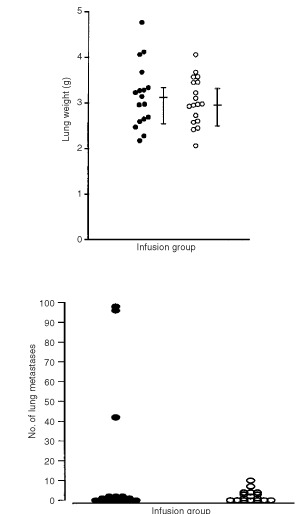
There was no significant difference (M.W.U. *P*=0.3) in the lung weights of animals with bFGF infused primary K12/TR tumours compared with controls (•: saline *n*=17; ○: bFGF *n*=18; median and interquartile range) (above). There was also no significant difference (M.W.U. *P*=0.3) in the number of lung metastases per animal with bFGF infused primary K12/TR tumours compared with controls (•: saline *n*=17; ○: bFGF *n*=18; median and interquartile range) (below).

Thirty-five animals were studied (saline *n*=17 and bFGF *n*=18). There was no significant difference in animal weights (*P*=0.2) between bFGF (421 g (309–432 g)) and saline groups. There was also no significant difference in lung weight (*P*=0.3, Figure 4

) or in number of lung metastases per animal (*P*=0.3, Figure 4) between bFGF and saline infused groups. Grossly enlarged ipsilateral axillary lymph nodes were identified in three (two bFGF infused and one saline infused) of the 35 animals studied. Metastases were not identified in other organs.

### Systemic bFGF infusion in animals bearing K12/TR flank tumours

#### Vascularity, blood flow, proliferation and growth

Ten tumours (five saline and five bFGF) from 10 animals were examined. There was no significant difference (*P*=0.4) in animal weights between bFGF (329 g (309–421 g)) and saline (319 g (298–398 g)) groups.

**Figure 5:**
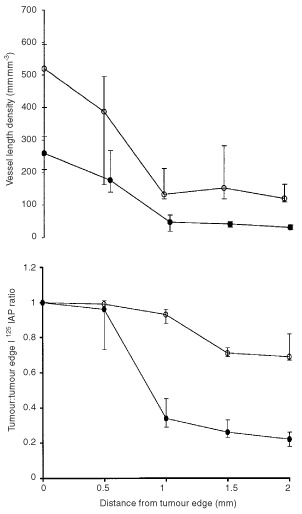
There were significant increases with distance from the tumour edge in vessel length density (repeated measures analysis of variance, *P*=0.0001) (above) and tumour to tumour edge blood flow ratio (*P*=0.0001) (below) in subcutaneous K12/TR tumours infused systemically with bFGF compared with controls (•: saline *n*=5; ○: bFGF *n*=5; median and interquartile range).

There was a significant increase (*P*=0.009) in vessel length density in the bFGF (median 36.2 mm mm^−3^, iqr 30.8–56.2 mm mm^−3^) compared with the saline-infused (10.6 mm mm^−3^, 9.8–20.0 mm mm^−3^) group. Similarly, there was a significant increase (*P*=0.009) in vessel volume fraction in the bFGF (6.4%, 5.5–7.3%) compared with the saline-infused (1.8%, 1.3–2.0%) group. There was a significant increase (*P*<0.0001) in vessel length density with distance from the tumour edge between bFGF and saline-infused tumours (Figure 5

). There was also a significant increase in tumour blood flow (*P*<0.0001) with distance from the tumour edge between bFGF and saline-infused tumours (Figure 5).

**Figure 6:**
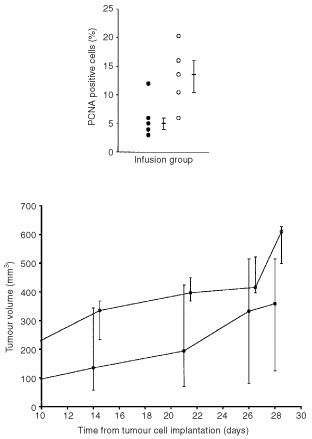
There was a significant increase (M.W.U. *P*=0.04) in the PCNA index in subcutaneous K12/TR tumours infused systemically with bFGF compared with controls (•: saline *n*=5; ○: bFGF *n*=5; median and interquartile range) (above). There was no significant increase (M.W.U. *P*=0.08) in the tumour volume of systemic bFGF infused subcutaneous K12/TR tumours compared with controls (•: saline *n*=5; ○: bFGF *n*=5; median and interquartile range) (below).

There was a significant increase in PCNA index (*P*=0.04) in the bFGF (median 13.6%, iqr 10.4–16.0%) compared with the saline-infused (5%, 4.0–6.0%) group. There was a trend that did not reach statistical significance (*P*=0.08), for an increase in tumour volume of bFGF compared with saline-infused tumours (Figure 6

).

#### Lung metastasis

**Figure 7:**
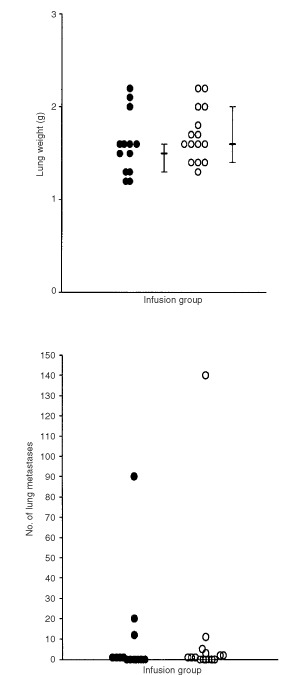
There was no significant difference (M.W.U. *P*=0.2) in the lung weights of animals with primary K12/TR tumours infused systemically with bFGF compared with controls (•: saline *n*=13; ○: bFGF *n*=15; median and interquartile range) (above). There was no significant difference (M.W.U. *P*=0.9) in the number of lung metastases per animal with primary K12/TR tumours infused systemically with bFGF compared with controls (•: saline *n*=13; ○: bFGF *n*=15; median and interquartile range) (below).

Twenty-eight animals were studied (systemic saline infusion *n*=13, and systemic bFGF *n*=15). There was no significant difference (*P*=0.4) in animal weights between bFGF (421 g (313–439 g)) and saline (413 g (304–459 g)) groups. There was also no significant difference in lung weight (*P*=0.2, Figure 7

), or in number of lung metastases per animal (*P*=0.9, Figure 7) in bFGF compared with saline-infused groups. Enlarged ipsilateral axillary lymph nodes were identified in five (three bFGF infused and two saline infused) animals. Metastases were not detected in other organs.

## DISCUSSION

The finding that bFGF increased *in vitro* K12/TR adenocarcinoma proliferation suggests that K12/TR proliferation was sensitive to bFGF. This is likely to have been mediated by bFGF receptors that have been described on the cell surface of some colonic adenocarcinomas (Terayama *et al*, 1996). This was consistent with the significant *in vivo* increases in flank tumour proliferation and growth that occurred with bFGF infusion.

Intratumoural and systemic bFGF infusion increase tumour vascularity and blood flow in both the HSN sarcoma and the K12/TR adenocarcinoma cell lines (Davies *et al*, 2001). Since the HSN cell line does not proliferate in response to *in vitro* bFGF or grow more rapidly *in vivo* on exposure to bFGF (Mathur *et al*, 1999), it is most likely that the bFGF-related tumour vascularity response was independent of tumour type – for example deriving from a bFGF effect on host endothelial cells. This supports the hypothesis that endothelial cells and tumour cells are separate tumour components that can be individually manipulated (Folkman, 1996).

Interstitial bFGF infusion approximately doubled K12/TR flank tumour vascularity, blood flow, proliferation and growth. These increases were compatible with the primary tumour vascularity increases that have been clinically associated with an increased risk of metastasis (Weidner *et al*, 1991; 1993). It was unlikely that bFGF administered into the interstitial space around the flank tumour reached the lung metastatic site. The absence of a detectable increase in lung metastasis suggested that these changes in flank tumour vascularity and blood flow did not produce a 50% increase in lung metastasis.

Systemic bFGF infusion produced increases in flank tumour vascularity, blood flow and growth that were of a similar magnitude to those achieved with interstitial infusion of a 50-fold smaller bFGF dose. Thus systemically-administered bFGF was active at the flank tumour site. Despite systemic bFGF levels that were capable of increasing flank tumour vascularity and can be expected also to have reached the lungs, there was no significant increase in lung metastasis.

These results do not support a role for raised levels of bFGF at either the primary or metastatic tumour site, or for associated increases in flank tumour vascularity and blood flow, in increasing metastasis. Even if increased primary tumour vascularity increased vascular intravasation (Liotta *et al*, 1974) – which is controversial (Ennis *et al*, 1997; Wharton *et al*, 1999; Mathur *et al*, 2001) – metastasis also involves additional critical steps – including attachment to vascular endothelium, and extravasation (Fidler, 1997). These steps may also have to be up-regulated to enhance tumour metastatic phenotype. The results were consistent with the clinical association between tumour vascularity and metastasis arising as consequences of a tumour genotype – for example a k-ras or p53 (Rak *et al*, 1995; Bartsch *et al*, 1998; Maehara *et al*, 2000; Mehta *et al*, 2001) mutation – that associated increased tumour vascularity with metastatic phenotype.
